# Semen adaptation to microbes in an insect

**DOI:** 10.1093/evlett/qrae021

**Published:** 2024-05-20

**Authors:** Oliver Otti, Natacha Rossel, Klaus Reinhardt

**Affiliations:** Animal and Plant Sciences, University of Sheffield, Sheffield, United Kingdom; Animal Population Ecology, Animal Ecology I, University of Bayreuth, Bayreuth, Germany; Applied Zoology, TU Dresden, Dresden, Germany; Animal and Plant Sciences, University of Sheffield, Sheffield, United Kingdom; Animal and Plant Sciences, University of Sheffield, Sheffield, United Kingdom; Applied Zoology, TU Dresden, Dresden, Germany

**Keywords:** coevolution, fecundity, host–parasite interactions, speciation

## Abstract

Sperm function is suggested to evolve by sexual selection but is also reduced by microbial damage. Here, we provide experimental evidence that male fertility can adapt to microbes. We found that in vivo, male fertility was reduced by one-fifth if sperm encountered microbes in the females that they had not previously been exposed to, compared to sperm from males that coevolved with these microbes. The female immune system activation reduced male fertility by an additional 13 percentage points. For noncoevolved males, fertility was larger if microbes were injected into females after they had stored away the sperm, indicating microbial protection as a previously unrecognized benefit of female sperm storage. Both medical and evolutionary research on reproductive health and fertility will benefit from considering our findings that the impact of microbes on sperm depends on their joint evolutionary history. Our results may assist in reconciling contradictory results of sexually transmitted disease effects on sperm and bring empirical realism to a recently proposed role of locally adapted reproductive microbiomes to speciation.

Sperm function is central to male fertility. It evolves by male and female genetics (Birkhead et al., 2009; Eberhard, 1996; Evans et al., 2019; Firman et al., 2017; Parker, 1970, 2014) and environmental variation (Evans et al., 2019; Reinhardt et al., 2015), modulated by seminal fluid (Perry et al., 2013; Poiani, 2006). Recent medical and evolutionary literature shows strong evidence for microbial effects on sperm function (Altmäe et al., 2019; Reinhardt et al., 2015; Rowe et al., 2020), suggesting that microbes could be an important driver of male fertility, male fitness, and thus, evolutionary change. Microbes can interfere with sperm directly. Sperm might encounter sexually transmitted microbes during or after mating (e.g., Afzelius et al., 1989; Eley et al., 2005b; Lockhart et al., 1996). Even opportunistic microbes on genitalia (Marius-Jestin et al., 1987) may be able to enter the reproductive tract via genital openings or copulatory wounds during mating (Bellinvia et al., 2020c). Their sperm will be exposed to the rich microbial flora of the female genital tract (e.g., Hirsh, 1999; Virecoulon et al., 2005). Selection for reproductive success before and after mating, including selection on sperm numbers, motility, fertility, and sperm competition, is covered by sexual selection theory (Birkhead et al., 2009; [Bibr CIT0047][Bibr CIT0048]). Precopulatory sexual selection includes microbes and parasites as selective agents (Hamilton & Zuk, 1982). However, whether microbial attacks on gametes adaptively shape male fertility after copulation has not been addressed by this body of theory on adaptive evolutionary change.

Host–parasite coevolution shows that pathogen defense is also a fundamental driver of adaptation. Microbial infections reduce host physiological functions, health, lifespan, and fitness (Schmid-Hempel, 2011). Microbial damage, costs of immune responses, and host resistance jointly shape the host’s evolutionary response to pathogens. Gametes, especially sperm, are frequently exposed to microbes and experience damage from microbes (Eley et al., 2005a; Otti et al., 2013). Consequently, sperm producers are expected to be under selection to produce ejaculates resistant to microbial damage (Otti, 2015; Reinhardt et al., 2015; Rowe et al., 2020). The geography of host–parasite coevolution then predicts that ejaculate-microbe interactions to lead to locally adapted ejaculates, a prediction that has fundamental significance also for sexually transmitted infections.

Here we apply this fundamental prediction of host–parasite theory to sperm biology and test whether male fertility evolves via ejaculate adaptation to microbial damage. If the male ejaculate adapts to microbial damage and affects sperm function, then males are expected to have greater fertility if their sperm are attacked by microbes that sperm coevolved with, compared to attacks by microbes that are novel to males and their sperm. Demonstrating adaptation requires the explicit test that male fertility directly affects the female’s reproductive output, or more precisely male fitness, and to separate the microbial effects on fertility from other effects of the infection in vivo, such as immune activation or resource allocation. Our model system, the common bedbug, *Cimex lectularius,* allowed us to do so and confirm the fundamental coevolutionary prediction that male fertility adapts to microbes. Male bedbugs traumatically inseminate females by piercing a hole through the abdominal cuticle of the female (Usinger, 1966). The copulatory organ of males carries a predictable community of microbes—that of the immediate environment - (Bellinvia et al., 2020a, 2020b; Otti et al., 2013, 2017; Reinhardt et al., 2005), which are introduced into the female body during mating (Bellinvia et al., 2020a, 2020b, 2020c; Otti et al., 2013, 2017). The community of microbes mainly includes the bacterial genera *Staphylococcus*, *Bacillus*, and *Acinetobacter* (Otti et al., 2017). Despite some antibiotic rescue by the ejaculate, the transferred microbes kill 40% of sperm in vitro (Otti et al., 2013). After mating, sperm pass through the female body cavity over several hours before being stored in special organs (Carayon, 1966). A single mating can provide enough sperm to fertilize eggs for up to 10 weeks (Reinhardt & Ribou, 2013).

We harnessed several aspects of the unusual traumatic insemination to establish an experimental injection system (Reinhardt et al., 2003) that controls for any impact of wounding on sperm or on the female (using surface-sterilized males as controls) and for any immune system activation (using the injection of heat-killed bacteria as a control). We injected local microbes into females after they had mated with surface-sterilized males. Using males from two populations, we addressed the potential for local adaptation of sperm to microbes. One population (population A) had coexisted with the local microbe community in our laboratory (environment A) for 12 years, a long-term co-exposure that may have led to A sperm adapting to environment A microbes. The other population (B) had been cultured in our laboratory for only 2 years and, therefore, experienced less exposure and B sperm had less time to adapt to environment A microbes. Microbial damage to sperm is commonly tested in vitro (Diemer et al., 1996, 2000; Eley et al., 2005a; Otti et al., 2013; Puerta Suarez et al., 2017; Reinhardt et al., 2015). Testing microbial damage to sperm in the female in vivo is desirable but difficult because the microbial injection will affect not only the sperm inside the female but also the female physiology itself, as well as the wounding and wound repair (Archie, 2013; Plaistow et al., 2003; Reinhardt et al., 2003; Theopold et al., 2004) elicited by the experimental infection. Finally, other immune system responses (Boltaña et al., 2013; Elliot et al., 2005; Kluger, 2015; Råberg et al., 1998; Sadd & Siva-Jothy, 2006; Stojanovich & Marisavljevich, 2008) and female resource allocation to defense (Lawniczak et al., 2007; Moret & Schmid-Hempel, 2000; Schwenke et al., 2016), sometimes concurrently (Radhakrishnan & Fedorka, 2012), can have large effects on sperm in the female and potentially mask the isolated microbial effects. We, therefore, disentangled female wounding and immune response effects on sperm from the effects of microbes. Finally, we carried out the infection at two-time points. Infecting females 6 hr after mating meant that microbes directly meet the sperm that then freely resides in the female hemolymph. Infecting another group of females 24 hr after mating meant that females would undergo similar resource allocation but sperm were stored away in the female storage organs, an indirect exposure to microbes.

## Results and discussion

**Figure 1.**
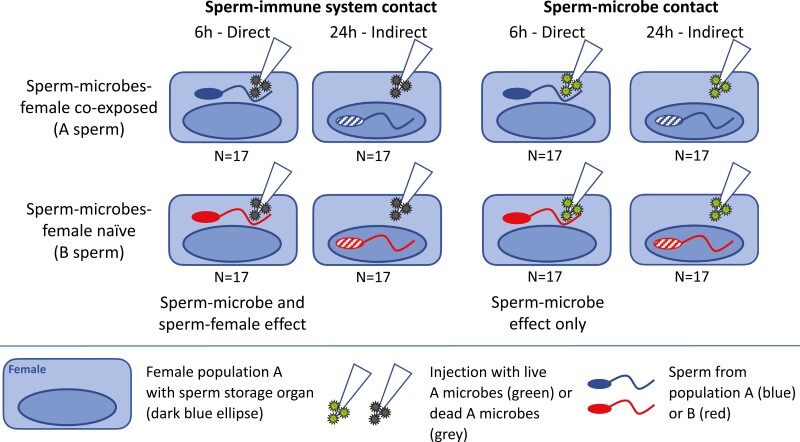
Simplified version of the experimental design used to disentangle female effects caused by microbe effects on sperm vs. immune system effects on sperm, as well as separating female sperm from microbe-sperm co-exposure. Untreated control (*n* = 33) and wounding control (*n* = 67) females are not presented graphically here. Note that immune system activation includes a separate treatment level, wounding only, subsumed here as an injection without live microbes (white arrows with gray stars at the tip representing dead microbes). A unique advantage of our study system is that fertility effects that arise from female resource allocation after infection can be controlled experimentally: An experimental infection before sperm storage (6 hr after mating, “direct treatment”) includes microbial impacts on the sperm plus collateral immune damage plus fertility reduction due to female defense allocation costs. Experimentally challenging females after sperm had been moved to sperm storage organs (24 hr after mating, “indirect treatment”) separates sperm from microbes and immune effectors present in the female hemolymph and only causes female defense allocation effects on fertility. Therefore, fitness costs of a microbial attack on sperm before storage (“direct treatment”) consist of (i) direct microbial plus immune costs on sperm and (ii) female defense and resource allocation costs.

**Figure 2.**
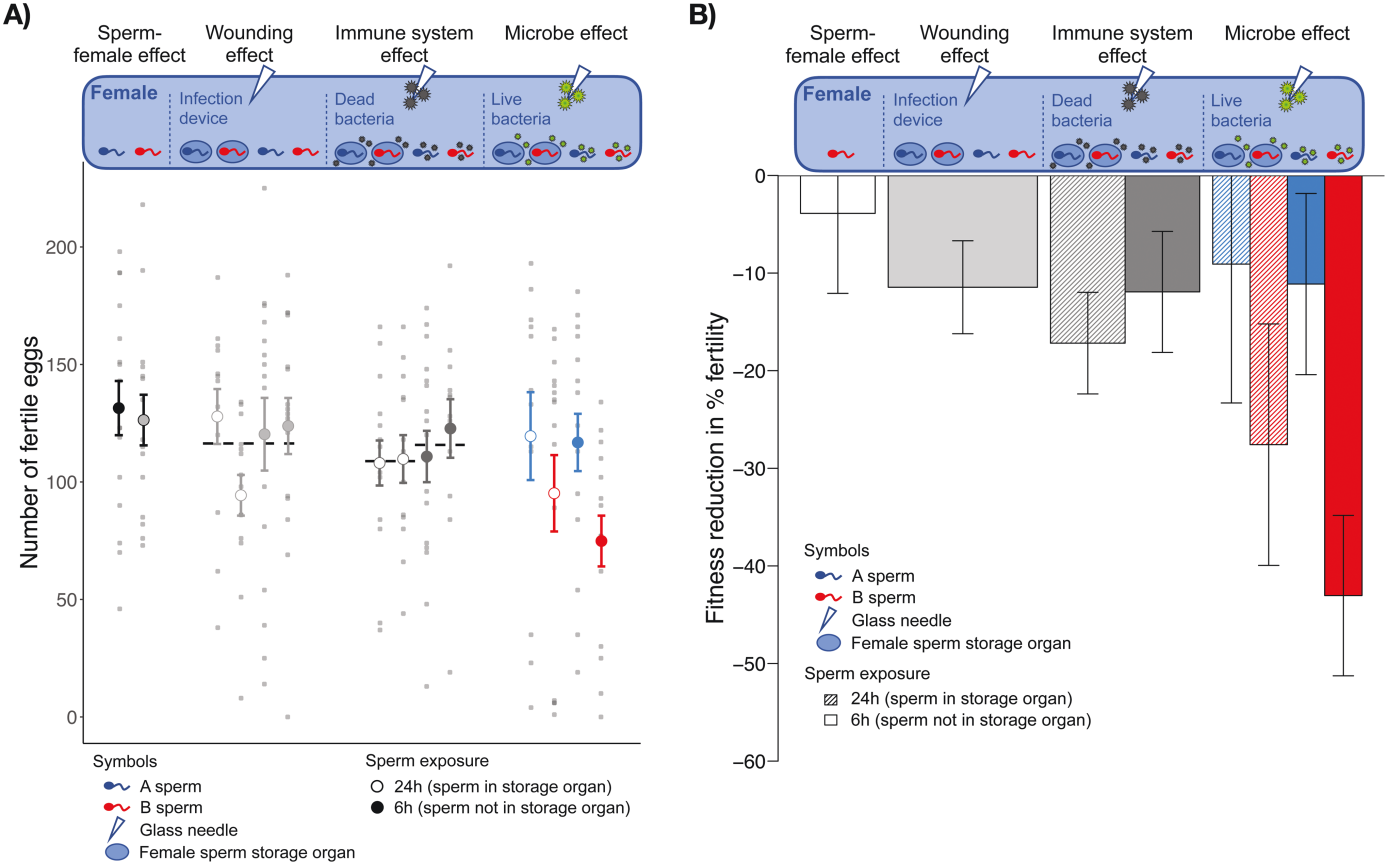
Female lifetime fertility in relation to infection treatments and microbe exposure of sperm. (A) Number of fertile eggs for the control (black), mating with a foreign male (very light gray), wounding controls (light gray), female immune system activation (gray), and microbial toxicity (A sperm in blue/B sperm in red). Sperm had either been directly exposed to microbes (“6 hr after mating”—filled coloration) or after they were in the female sperm storage organ (“24 hr after mating”—white circles). (B) Female lifetime fertility relative to the fertility of control females in relation to infection treatments and microbe exposure of sperm. Together, wounding, immune system activation, and microbial toxicity reduced fertility by 10% for co-exposed and by 35% for B sperm. The bars show the proportional decrease of lifetime fertility per female for mating with a foreign male (white), wounding only (light gray), female immune system activation (gray), and microbial toxicity (blue/red). Sperm had either been directly exposed to microbes (“6h after mating”—filled coloration) or after they were in the female sperm storage organ (“24h after mating”—hatched coloration). A fitness cost of a microbial attack on sperm before storage (6 hr after mating,” “direct treatment”) consists of (i) direct microbial plus immune costs on sperm and (ii) female defense and resource allocation costs. A microbial attack of females after sperm had been moved to storage (24 hr after mating, “indirect treatment”) only causes fitness costs of (ii). Deducting costs of (ii) from the total fitness costs [(i) and (ii)] will therefore produce the fitness costs (i), i.e., those arising from the direct microbial, or immune, impact on sperm. The average reduction in fertility caused by the immune system was 14.3% (mean of hatched and filled gray bars). The effect of microbial toxicity is only visible for direct contact (filled bars) and is much stronger if microbes and sperm are not coadapted. On average, B sperm caused a 36% reduction in female fertility (mean of hatched and filled red bars) compared to the mean number of eggs laid by untreated control females mated to males of their own population. The number below the error bars represents the absolute mean difference in the number of eggs per female. Error bars represent one standard error.

We performed a single large experiment and first separately presented the results of direct microbe-sperm contact, fully controlling for wounding and immune activation effects in the female (Figure 1, Material and Methods in Supplementary material, Supplementary Table S2, Supplementary Data S1—semen microbe adaptation.csv). Direct microbe-sperm contact refers to injecting live bacteria into females after females have mated but before the sperm has been stored. Extracting the effects of only live microbes, we confirmed our central prediction that the male ejaculate adapts to microbial damage affecting sperm function: We found that population B sperm had 21.2 percentage points (p.p.) lower fertility (number of fertilized eggs) when exposed to A microbes compared to A males exposed to A microbes in vivo (Supplementary Table S2: “net cost of microbial damage to B sperm”). This is an unusually large environmental effect on sperm (Reinhardt et al., 2015). Also supporting our central prediction, male fertility was not reduced in A sperm at all (Figure 2) when correcting for the 9.3 p.p. fertility reduction by wounding and immune system activation.

Details of how co-exposure history was separated from general microbial effects on sperm per se can be found in our full dissection of experimental effects on male fertility (Table 1; Supplementary Tables S1 and S2). Statistical modeling of all factor levels (Model 1 in Table 1) showed that co-exposure history explained 7.5% of the variation in male fertility (Model 1: GLM: *F*_1,198_ = 2.023, *p* < 0.05, Table 1). The largest part of this variation (83%) arose from contrasting the microbial BA sperm effects versus all other effects combined (Model 10: GLM: *F*_1,205_ = 12.075, *p* < 0.001, Table 1), again isolating sperm exposure to “novel” microbes as the major driver of male fertility in our experiment. In fact, any model that considered co-exposure history separately from general microbial effects on sperm performed much better than those not considering it (Table 1). These results are consistent with our central prediction, which is that the male ejaculate adapts to microbial damage that affects sperm function. Therefore, we propose to include ejaculate adaptation to microbes as a process of host–pathogen interactions. While it is well known that female fitness can be dramatically reduced by microbial infection (Schwenke et al., 2016), we show that more than half of the fitness costs of infection (21 p.p. of 35.5% total) in our system arose because “novel” microbes damaged sperm. This large effect provides reasons to suggest that future studies might benefit from dissecting infection costs into direct costs to females and to costs that arise from sperm damage.

**Table 1.** Model selection of female fertility as a function of treatment level. Models are sorted in ascending order by AICc.

Model	Treatment levels	*df*	logLik	AICc	deltaAICc	Weight
10	(C + F + W + ISIN + ISD + MIA + MDA), (**MIB + MDB)**	3	−1092.180	2190.479	0.000	0.342
7	(C + F + W + ISIN + ISD + MIA + MDA), **MIB, MDB**	4	−1091.465	2191.127	0.648	0.248
8	(C + F + W + ISIN + ISD + MIA + MDA + MIB), **MDB**	3	−1093.100	2192.318	1.839	0.136
9	(C + F + W + ISIN + ISD), **(MIA + MDA),(MIB + MDB)**	4	−1092.179	2192.556	2.077	0.121
6	(C + F + W + ISIN + ISD + MIA), **MDA, MIB, MDB**	5	−1091.462	2193.223	2.744	0.087
5	(C + F + W + ISIN + ISD), **MIA, MDA, MIB, MDB**	6	−1091.452	2195.324	4.845	0.030
11	(C + F + W + ISIN + ISD), (**MIA + MDA + MIB + MDB**)	3	−1095.612	2197.343	6.864	0.011
4	(C + F + W + ISIN), **ISD, MIA, MDA, MIB, MDB**	7	−1091.428	2197.420	6.941	0.011
3	(C + F + W), **ISIN, ISD, MIA, MDA, MIN, MDB**	8	−1090.755	2198.236	7.757	0.007
2	(C + F), **W, ISIN, ISD, MIA, MDA, MIB, MDB**	9	−1090.020	2198.953	8.474	0.005
1	**C, F, W, ISIN, ISD, MIA, MDA, MIB, MDB**	10	−1089.974	2201.070	10.591	0.002

*Note*. The models with the highest support from the data, as indicated by AICc weights, were models separating control, mating with foreign males, wounding, and female immune system effects from sperm coadaptation level and sperm-microbe contact. C = control; F = mating with foreign male; W = wounding; ISIN = indirect effect of female immune system activation; ISD = direct effect of female immune system activation; MIA = microbial effects indirect on A sperm; MDA = microbial effects direct on A sperm; MIB = microbial effects indirect on B sperm; MDB = microbial effects direct on B sperm.

In addition to the isolated microbial costs on male fertility, we found that wounding (7.5 p.p. fertility reduction) and female immune system activation by dead bacteria (1.8 p.p.) reduced sperm function (Figure 2B; Supplementary Table S2, Supplementary material). Again, previous reports of such costs to females (Ardia et al., 2012; McNamara et al., 2014; Moret & Schmid-Hempel, 2000) have not been able to separate costs on sperm function from costs on females. The possibility that B sperm themselves, which have not coevolved with females, impose a fitness effect is small: B sperm reduced female fitness differences by only 3.9% compared to A sperm (Figure 2B), similar to previous findings in the species (Reinhardt et al., 2009a).

The first part of the results arose from experimentally infecting females 6 hr after mating, when sperm resided in the hemolymph (Carayon, 1966). The second part of our results arose from experimentally infecting females 24 hr after mating, when sperm are stored in specialized organs, somewhat separated from microbes and immune effectors in the female hemolymph. Note that females thus treated will still show the fertility changes caused by resource allocation to immune function, defense, and repair, as seen in the 6 hr treatment. We found that microbial infection caused a significantly smaller fertility loss if B sperm had been stored during infection compared to B sperm residing in the female body cavity during infection (Figure 2). Because A sperm showed no such difference (Figure 2), that result revealed protection of sperm from “novel” microbes as a benefit of female sperm storage. This finding was unexpected and not previously predicted (Orr & Brennan, 2015). Because long-term female sperm storage is nearly ubiquitous among internally fertilizing species (Orr & Brennan, 2015) it will be interesting to test the generality of this benefit in other animals. Female insects have previously been shown to increase antimicrobial activity (Dávila et al., 2018) or immune gene expression (Chérasse et al., 2018; Delbare et al., 2020) in sperm storage organs. Our data suggest that future studies analyzing reproductive microbiomes (Altmäe et al., 2019; Rowe et al., 2020) will benefit from separating such responses between co-exposed and novel microbes.

**Figure 3.**
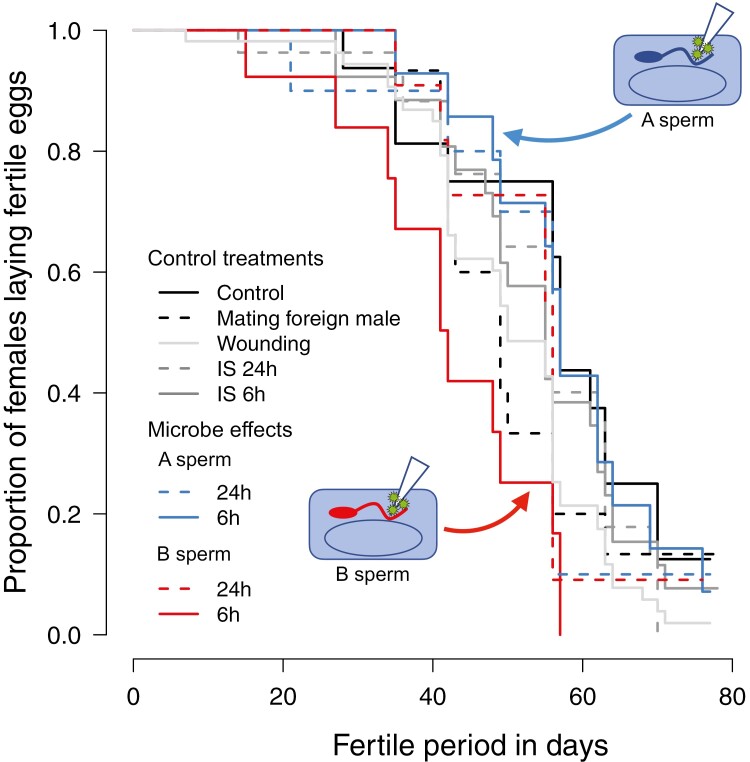
Proportion of females laying fertile eggs under control and microbial treatments. Females mating with their own males (control treatment): black solid line; females mating with a foreign male (dashed black line). Wounded control females (light gray line), control females immune-activated (IS indirect and direct) with dead microbes (gray lines), and the females injected with live microbes (A sperm blue and B sperm red lines). Females used sperm that were co-exposed (blue lines) or B sperm (red lines) to microbes used, and that experienced direct exposure (solid lines) or indirect (after storage) exposure (dashed lines). The probability of becoming infertile differed between treatments (Cox proportional hazards: Wald *χ*^2^_8,170_ = 15.420, *p* = 0.05). Females using B sperm had a 3.73× higher risk of becoming infertile than control females when sperm were directly exposed to microbes (Cox proportional hazards: *z* = 3.294, *p* < 0.001) but not when sperm had been stored in the seminal vesicles (1.7×; Cox proportional hazards: *z* = 1.258, *p* = 0.21). Wounding increased this risk by a factor of 1.9 (Cox proportional hazards: *z* = 2.076, *p* = 0.04). Effects of mating with a foreign male, direct and indirect immune system activation, and fertilization with co-exposed sperm after directly or indirectly encountering microbes were all nonsignificant.

Sperm storage provided the benefit of antimicrobial protection only for “novel” microbes (B sperm). We, therefore, predicted that microbial infection before, but not after, sperm storage would shorten the fertile period of females using B but not of females using A sperm. Our data support this assumption: Females using A sperm did not differ from controls in the length of the fertile period (Figure 3) but females using B sperm had almost a 4 times higher risk of becoming infertile (Cox proportional hazards: *z* = 3.294, *p* < 0.001) when infected after 6 h but not when infected after 24 h (Figure 3). This reduction in the fertile period amounted to an average of 15 days or 20%–25% of the lifespan (Figure 3 and Supplementary Figure S2).

The 20%–25% reduction of male fertility via a reduction of the female fertile period was similar to the overall fertility reduction of 21.2 p.p. in the same treatment (see above). Therefore, we tested whether male fertility decreased mainly because the females had a shorter fertile period or whether the rate of production of fertilized eggs also decreased. We found that male fertility was reduced due to decreased fertilization success (Tukey’s HSD test, direct B sperm vs. direct A sperm treatment: *p* < 0.03). Later in life, B sperm caused approximately 30% lower fertility rates per week than A sperm (LME: *F*_7,40.612_ = 18.234, *p* < 0.001) (Supplementary Figures S1 and S2). Such a decrease in fertilization success over time is consistent with fertility limitation by sperm aging (Pizzari et al., 2008; Reinhardt, 2007). As fertility limitation over time is stronger in B sperm, we speculate that sperm aging depends on the sperm’s environment. Because the sperm-microbe treatments produced similar patterns for fertility, i.e., the number of fertile eggs laid (Figure 1), and for fecundity, i.e., the total number of eggs laid (Supplementary Figure S3 and Supplementary Table S3), it seems microbial impacts on sperm caused females to lay fewer eggs, rather than to fertilize fewer eggs or to show reduced embryo development. This may happen if microbes kill sperm (Otti et al., 2013) rather than reducing the sperm’s fertilization ability, a speculation that may be tested in future experiments.

### Conclusion

Microbial impacts on sperm contribute to a considerable infection-related loss of male fertility, but only if sperm had little prior exposure to microbes. This is a strong footprint of selection on ejaculates to resist a microbial attack that provides a novel link of sexual selection with host–parasite interactions. Future studies should examine the effect of individual microbe species vs. microbial communities and how these affect the relative importance of microbial and immune system activation costs on sperm function. Our data only present the results from a single population, but they still highlight the importance of sperm-microbe interactions for evolutionary, ecological, and medical research. This significance should be tested in more populations and more species, as well as a wider range of infection and sperm competition scenarios. Considering the degree of co-exposure between sperm and microbes may help to explain contradictory results of microbe-induced sperm damage in the medical literature (e.g., Puerta Suarez et al., 2017 vs. Eley et al., 2005a; Diemer et al., 1996 vs. Diemer et al., 2000). Finally, the effect of microbes on foreign ejaculates supports the recent suggestion that microbes could act as a reproductive barrier in the speciation process (Rowe et al., 2020).

## Methods

### Bedbug culture and experimental rationale

#### Culture

Bedbugs were maintained at 26 ± 1 °C, at 70% relative humidity with a cycle of 12L:12D using the feeding, maintenance, and generation-of-virgin-individuals protocol of (Reinhardt et al., 2003). All individuals were virgins prior to the experiment. We used individuals from two large stock populations (>1,000 individuals) of different origins (arbitrarily called *A*, *B*) maintained in the laboratory for different amounts of time. Population *A* was of unknown origin in the wild but had been maintained at the University of Sheffield for ~12 years (ca. 100 bug generations) and before that for >40 years at the London School of Hygiene and Tropical Medicine. Population *B* was collected in Nairobi in 2008 and maintained at the University of Sheffield for 2 years prior to the experiments (less than 15 generations).

Females and microbes used in the experiment were all from population A, and sperm were from either population A or B. Therefore, when A or B males were mated with A females, the time for female-microbe coevolution was the same, but the time for sperm-microbe coevolution differed: 100 vs. 15 generations for A and B males, respectively.

### Mating treatment

All females (*n* = 236) were virgin and 14 days old at the time of mating. They were allocated to a single mating with a randomly drawn, 14-day-old male from either population *A* or *B* (for each *n* = 118). Matings were staged, monitored, and interrupted after 60 s as described in Reinhardt et al. (2009b). Matings were interrupted to standardize sperm number transferred because there is a linear relationship between copulation duration and sperm number (Siva-Jothy & Stutt, 2003). A standardized sperm number was desirable since spermatozoa trigger the release of an oviposition-stimulating hormone from the *corpora allata* (Davies, 1965), and this could potentially influence the results.

Before applying any treatment, males had their intromittent organs surface-sterilized by dipping the last two abdominal segments into 1% E-Toxa-Clean detergent (Sigma-Aldrich, UK) for 10 s. This procedure significantly reduced the number of colony-forming units (CFU) of bacteria on the intromittent organ (surface-sterilized: mean ± sd 1.8 ± 2.49 CFU, untreated: 15.37 ± 22.66 CFU) (ANOVA with Box-Cox-transformed data (*λ* = 0.095): *F*_1,39_ = 17.370, *p* < 0.01). The treatment had no effect on sperm viability or survival. All 10 treated and 10 untreated individuals survived at least 3 weeks. The proportion of dead sperm was analyzed using the LIVE/DEAD sperm viability kit (Invitrogen), as described in Otti et al. (2013). This proportion did not differ between the treated mean ± sd = 0.18 ± 0.19 and untreated males (0.15 ± 0.09) (generalized linear-mixed effects model with binomial distribution and male ID fitted as a random effect with five replicate measures on each of three males in each treatment: *χ*^2^ = 0.041, *df* = 1, *p* = 0.839). After surface sterilization, males were individually transferred to a sterile Petri dish (∅55 mm) containing an autoclaved filter paper disk and left to dry for 10 min. Then, they were mated with females within 30 min after surface sterilization.

### Infection treatments

Females mated to A or B males were randomly allocated in equal numbers (*n* = 59) to one of two microbial challenge treatments, the infection being administered 6 hr after mating or 24 hr after mating. Within these treatment groups, the second treatment was implemented, with equal numbers of females allocated to either of four levels of microbial treatment: (i) control (no treatment, total *n* = 33), (ii) wounding only (total *n* = 67), (iii) wounding plus immune system activation (total *n* = 68) and (iv) wounding plus immune activation plus infection total (*n* = 68) (Figure 1). Costs of the microbial challenge 2–10 hr after mating represent microbial and immune system costs on females plus microbial and immune system costs on the sperm they had received (“direct treatment”). The costs of the microbial challenge 24 hr after mating mainly include microbial and immune system costs to females (“indirect treatment”). This infection treatment isolated costs of a microbial challenge to free sperm from costs on females plus costs on stored sperm (see also Main text and Figure 1). Control females were handled identically to all other females except for treatment administration. Females subjected to wounding were pierced into the intersegmental membrane between the 2nd and 3rd abdominal sternites using a sterile, autoclaved glass needle pulled to a fine point. Prior to use, females were placed on ice for 30 min for immobilization. Thereafter, females were allowed to recover for four hours and were then fed. Wounding, plus the activation of the immune system, was administered by piercing the females with a glass needle, as described above. However, the autoclaved needle was first dipped in a solution of heat-killed microbes (see *Microbe solution* section), left to dry, and then used for piercing. The wounding plus immune activation plus infection effects were imposed by piercing females with a glass needle that, after autoclaving, was dipped into a solution of live bacteria (see *Microbe solution*). Immobilization on ice, piercing, and feeding was as described for the wounding treatment. During the infection treatment, the experimenter was unaware whether females had received co-exposed or B sperm and, hence, blind to our main experimental treatment. Also, egg counts were recorded blind.

### Microbe solution

Following an earlier protocol that examined microbe effects on sperm, we produced a microbe solution by soaking a filter paper from population A for 2 hr at room temperature in phosphate-buffered saline (PBS: 8.74% NaCl, 1.42% Na_2_HPO_4_, pH = 6.5) (Otti et al., 2013). The microbe solution was divided into two equal parts (10 ml). 10 ml were immediately aliquoted into 0.5 μl Eppendorf tubes and stored at minus 80 °C until use (live microbe solution). The other 10 ml was first autoclaved for 15 min at 121 °C, then aliquoted and frozen in the same way (dead microbe solution). On the day of experimentation, aliquots were thawed on ice and diluted 10 times in PBS. Previous data indicate the presence of at least 10 environmental microbial species that are present on the male genitalia and after mating in the female (Bellinvia et al., 2020a, 2020b; Otti et al., 2013, 2017; Reinhardt et al., 2005). The most common species found in the environment of the stock population belong to the genera *Staphylococcus*, *Bacillus*, and *Acinetobacter* (Otti et al., 2017). As the relative contribution of each microbial species to sperm mortality is not known, and it is unknown whether microbial effects are caused by individual species or by the whole community, we decided to use the broad spectrum of bacteria present (for a list of species see Table 1 in Otti et al., 2017).

### Female fitness

After mating and infection treatment, females were kept individually in 15 ml plastic tubes with a piece of filter paper for egg laying. Females were fed weekly, and the number of fertile and unfertilized eggs was counted weekly. Fertile eggs are turgid and whitish, with visible eye spots of the developing embryo. Unfertilized eggs normally collapse soon after being laid and are grayish. We measured two fitness parameters directly linked to sperm function, fertility, and fecundity. Fertility was the number of fertile eggs per female, and fecundity was the total number of eggs per female. Fertile and unfertilized eggs were distinguished, and the onset of fertilization senescence was defined (following Reinhardt et al., 2009a) as the time point when the second unfertilized egg was laid to allow for one accidental fertilization failure.

While bacteria are cleared from the hemolymph within a few days (Haine et al., 2008), several studies nevertheless show late-life effects of early infections (Finch, 2010; Khan et al., 2017; Pursall & Rolff, 2011). Therefore, we compared the effect of microbial infection on fertility and fecundity in the first 4 weeks after mating when no reproductive senescence effects occur (Reinhardt et al., 2009a) with that between weeks 5 and 10. Like this, we assessed whether any resource allocation to immunity during female reproductive aging would accelerate or alleviate the sperm effects (Supplementary Figure S2).

### Statistical analyses

All statistical analyses were performed using R 4.3.2 (R Core Team, 2023) and the package *MuMIn* (Bartoń, 2023). First, we developed 10 models fitting different treatment level combinations as factors to analyze effects on fertility according to our hypotheses. The first model incorporated the complete set of treatment levels, i.e., control, mating with a foreign male, effect of wounding, immune system activation, and microbial effects for both B sperm and co-exposed sperm, and for indirect and direct sperm effects (Table 1). We combined treatment levels one by one from the first to the last treatment level to test the best predictor. Models 9 and 10 were built by combining the treatment levels: indirect vs direct sperm effects, microbe presence vs. absence, and co-exposed vs. B sperm (Table 1).

Second, we fitted each of those factors using a generalized linear model (GLM) with the fertile number of eggs as a response variable. This led to 10 GLMs, from which we then ranked models using model selection based on Akaike’s Information Criterion, corrected for sample size (AICc; Akaike, 1973) (Table 1).

Twenty-nine females did not lay eggs and were removed from further analysis (control: 2, wounding: 7, immune activation: 11, microbial effects: 9). This biased the design towards a larger sample size of the control but still resulted in similar sample sizes between infection steps. We measured body size using pronotum width. Total egg numbers were significantly positively correlated with female body size (Pearson’s correlation: *r*^2^ = 0.173, *t* = 2.384, *df* = 184, *p* < 0.05), but female body size was not considered further because it did not differ between treatments (ANOVA: sperm genotype: *F*_1,185_ = 0.062, *p* = 0.80; microbe contact: *F*_1,185_ = 0.284, *p* = 0.59; steps of infection: *F*_3,185_ = 1.414, *p* = 0.24). Male body size did not differ between treatments (ANOVA: microbe contact: *F*_1,187_ = 0.384, *p* = 0.54; steps of infection: *F*_1,187_ = 0.298, *p* = 0.83). Males were significantly larger than B males (ANOVA: *F*_3,187_ = 11.593, *p* < 0.001), but male body size was not correlated to total egg numbers (Pearson’s correlation: *r*^2^ = −0.047, *t* = −0.640, *df* = 186, *p* = 0.52).

Package *survival* (Therneau, 2023) was used to conduct survival analyses on the probability of laying unfertilized eggs and a reduction of the sperm fertile period. For the analysis of the probability of laying unfertilized eggs, the second unfertilized egg laid represented the onset of infertility (Reinhardt et al., 2009a), which was used as the time variable in the survival function. We fitted a Cox proportional hazard model to investigate the effects of control, mating with a foreign male, effect of wounding, indirect effect of female immune system activation, direct effect of the immune system activation, indirect effect of microbes on A sperm, direct effect of microbes on A sperm, indirect effect of microbes on B sperm, and direct effect of microbes on B sperm (Table 1). To validate the model, we used the *cox.zph()* function. We used the package *multcomp* (Hothorn et al., 2008) for multiple comparisons, adjusting *p*-values using the Benjamini–Hochberg procedure (Benjamini & Hochberg, 1995). For the comparisons of early vs. late fertility and fecundity, we analyzed only the egg counts from the microbe toxicity treatments. We calculated an early and late egg-laying rate by averaging the egg counts from week 1 to 4 and week 5 to 10, respectively, to test whether male fertility was reduced mainly because the fertile period in females was shortened or whether decreases in the number of fertilized eggs also contributed. Then, we fitted linear mixed effects models with fertile and total egg numbers as response variables and a factor with eight levels representing each treatment combination of egg-laying phase, microbe exposure, and microbe-sperm adaptation. As a random effect, we fitted the female ID. Subsequently, we used the package *multcomp* (Hothorn et al., 2008) for multiple comparisons, adjusting p-values using the Benjamini–Hochberg procedure (Benjamini & Hochberg, 1995).

## Supplementary material

Supplementary material is available online at *Evolution Letters*.

qrae021_suppl_Supplementary_Material

## Data Availability

All data and R code are available in the main text or the supplementary materials (Otti et al., 2024 Data S1—semen microbe adaptation.csv and Otti et al., 2024 R script.R).
