# Correction to: Semen adaptation to microbes in an insect

**DOI:** 10.1093/evlett/qrae026

**Published:** 2024-06-14

This is a correction to: Oliver Otti, Natacha Rossel, Klaus Reinhardt, Semen adaptation to microbes in an insect, *Evolution Letters*, 2024, qrae021, https://doi.org/10.1093/evlett/qrae021

In the originally published version of this article, the first sentence was incorrectly given as follows:

“females laying fertile eggs under control Sperm function is central to male fertility.”

The Publisher apologizes for this error, which occurred during the production process.

The sentence has been corrected to read: “Sperm function is central to male fertility.”

