# Supplementary material for: Semen adaptation to microbes in an insect
Source: Evol Lett. 2024 May 20;8(5):638–46. doi: 10.1093/evlett/qrae021 (PMC11424074; doi:10.1093/evlett/qrae021)
Supplement: qrae021_suppl_Supplementary_Material [file qrae021_suppl_supplementary_material.zip › Otti.etal - semen adaptation to microbes - supplemental information.docx]

Supplemental information for

**Semen adaptation to microbes in an insect**

Oliver Otti, Natacha Rossel, Klaus Reinhardt

Correspondence to: [oliver.otti@tu-dresden.de](mailto:oliver.otti@tu-dresden.de)

**This file includes:**

Supplemental material and methods and results

Figs. S1 to S3

Tables S1 to S3

Captions for Data S1

**Other supplemental information for this manuscript includes the following:**

Data S1 - semen microbe adaptation.csv

R script Otti et al. 2024

Supplemental Material and Methods

**Bedbug biology and experimental rationale**

Naturally, sperm and microbes are deposited in a female paragenital organ, the mesospermalege. This organ already has some immune function and possibly digests sperm. Addressing the mesospermalege effects would have doubled the necessary sample size and made another treatment necessary. We decided against addressing these effects to keep our already complicated design manageable. Also, like this our results are conservative because only those sperm that even passed the mesospermalege were exposed to a microbial treatment.

**Complete dissection of the fitness costs on sperm**

We calculated the net fitness costs separately for wounding, female immune response and microbe damage depending on sperm-microbe interaction and co-exposure history. For this we used the means for each treatment and the fitness of the control mating of A female with A males. To assess the costs associated with foreign ejaculates, we subtracted the fitness of A female mating with a foreign male from the fitness of the control mating (A females with A males) (cost #2). To isolate the wounding effect, we subtracted control or cost #2 from the mean wounding effect (cost #3 for A, cost #4 for B). To calculate the isolated costs of the immune response, we subtracted costs #3 from the cost of immune activation for direct (cost #6) or indirect (costs #5) challenge of A ejaculates or direct (cost #8) and indirect (cost #7) challenge of B ejaculates. Finally, from the total fitness costs after microbial challenge on sperm, we subtracted the wounding and immune response costs from the microbial effect for the direct and indirect sperm-microbe interaction and for B sperm and A sperm (costs #9, #10, #11, #12)(Table S1). By averaging the fitness costs with their categories, we were able to calculate mean net fitness effects of female immune response and microbial damage on sperm depending on direct or indirect contact and co-exposure history (Table S2). In the following, we numbered the different contrasts of costs for the ease of understanding the net fitness effects in table S1 (costs: positive values; benefits: negative values).

Supplemental Results

**Fecundity and infection**

Similar to lifetime fertility, the best model indicated that microbial co-exposure affected the microbial toxicity impact on sperm fitness. Fitness was reduced by 35% for B sperm compared to 10% reduction in co-exposed sperm (Fig. S3). The best model separating the fitness effect of microbes on B sperm from all other effects was significant (GLM: F_1,205_=9.733, P<0.01) and received 1.5 times the support of the second-best model separating direct and indirect microbial toxicity on B sperm from all other effects (Ratio AICc weights: 0.32/0.22=1.5)(Table S3). A model distinguishing direct and indirect microbial effects on sperm showed a 39% fitness reduction for direct, and an 9% reduction for indirect effects after exposure to A microbes (1.9 times higher support than a model only distinguishing between microbial effects on B sperm and A sperm, Ratio AICc weights: 0.22/0.11=2)(Table S3). The third-best model, contrasting direct effects on BA sperm from all other treatments got a higher support than a model only contrasting the degree of sperm-microbe adaptation (Ratio AICc weights: 0.19/0.11=1.7)(Table S3).

The effect of the sperm genotype *per se*, rather than its coevolution state, had no effect on female fecundity, neither did wounding nor the activation of the immune system son sperm fitness. Models separating the effect of female immune system activation and wounding on sperm fitness, on top of any possible resource allocation by the female following the infection, received very low support (all AICc weights below 0.1) and considering direct immune effects (10% fertility reduction per female; Fig. S3) or indirect female immune effects (17% fertility reduction per female) weakened the model support even further (Ratio AICc weights: 0.01/0.03=0.33; Figure S2)(Table S3).

**Fig. S1**

**Figure S1** Mean number of early (closed symbols) and late (open symbols) fertile eggs per week over all combinations of microbe contact and co-adaptation. Individual numbers of fertile eggs represented by different colors and shapes: for microbial toxicity (A sperm – blue/B sperm - red) with respect to whether sperm were directly exposed to microbes (direct - triangles) or when in the female sperm storage organ (indirect – circles). Error bars represent one standard error.

**Fig. S2**

**Figure S2** Mean number of early (closed symbols) and late (open symbols) eggs per week over all combinations of microbe contact and co-adaptation. Individual numbers of fertile eggs represented by different colors and shapes: for microbial toxicity (A sperm – blue/B sperm - red) with respect to whether sperm were directly exposed to microbes (direct - triangles) or when in the female sperm storage organ (indirect – circles). Error bars represent one standard error.

**Fig. S3**

**A)**

**B)**

**Figure S3** Female lifetime fecundity in relation to infection treatments and microbe exposure of sperm. **A)** Total number of eggs for the control (black), mating with a foreign male (very light grey), wounding controls (light grey), female immune system activation (grey) and microbial toxicity (blue/red). Sperm had either been directly exposed to microbes ('6h after mating' - filled coloration) or after they were in the female sperm storage organ ('24h after mating' – white circles). **B)** Female lifetime fecundity in relation to infection treatments and microbe exposure of sperm. The bars show the proportional decrease of lifetime fecundity per female for mating with a foreign male (white), wounding only (light grey), female immune system activation (grey) and microbial toxicity (blue/red) with respect to whether sperm were directly exposed to microbes ('6h after mating' - filled coloration) or when in the female sperm storage organ ('24h after mating' – hatched coloration). The effect of microbial toxicity is only visible for direct contact (filled bars) and is much stronger if microbes and sperm were not coadapted. The proportional reduction refers to treatment deviations from the mean number of eggs laid by untreated control females, mated to females of their own population. The number below the error bars represents the absolute mean difference in the number of eggs per female. Error bars represent one standard error.

**Table S1** Mean fitness cost on sperm with respect to wounding, female immune response and microbe damage depending on sperm-microbe interaction and co-exposure history. Positive values are costs and negative values benefits. We calculated male fertility effects of co-exposure history by sequentially subtracting from the baseline fertility effects arising from i) mating with a male from the low-exposed population, ii) wounding, iii) female immune response, separately for the direct and indirect treatment. Contrasting these fitness effects to each other allowed us to deduce the net effects on male fertility via sperm of the female immune response and microbes depending on sperm storage and co-exposure history (Table S2).

| **Type of cost** | **Calculation** | **Fitness reduction in fertility given as percentage points** |
| --- | --- | --- |
| **Mating** |  |  |
| Control mating with A male: | | 0 |
| Mean cost #2) | Mating with B male: | 3.88% |
| **Wounding** |  |  |
| Mean cost #3) | Wounding minus control: | 11.31%-0% = 11.31 |
| Mean cost #4) | Wounding minus cost #2: | 11.31%-3.88% = 7.43 |
| **Immune system activation** | |  |
| Mean cost #5) | Immune response costs after mating with A male (A sperm, 24h) minus cost #3: | 17.8%-11.31% = 6.49 |
| Mean cost #6) | Immune response costs after mating with A male (A sperm, 6h) minus cost #3: | 15.7%-11.31% = 4.39 |
| Mean cost #7) | Immune response costs after mating with B male (B sperm, 24h) minus cost #4: | 16.5%-7.43% = 9.07 |
| Mean cost #8) | Immune response costs after mating with B male (B sperm, 6h) minus cost #4: | 6.61%-7.43% = -0.82 |
| **Microbial costs *per se*** |  |  |
| Mean cost #9) | Total costs minus cost #5: | 9.08%-6.49% = 2.59 |
| Mean cost #10) | Total costs minus cost #6: | 11.1%-4.39% = 6.71 |
| Mean cost #11) | Total costs minus cost #7: | 27.6%-9.07% = 18.53 |
| Mean cost #12) | Total costs minus cost #8: | 43.0%+0.82% = 43.82 |

**Table S2** The net effect of microbial exposure on sperm fertility potential while controlling for female immune activation, wounding, and sperm storage. Positive values indicate fitness costs, negative values indicate benefits. By contrasting the fitness effects from table S1 to each other allowed us to deduce the net effects on male fertility via sperm of the female immune response and microbes depending on sperm storage and co-exposure history.

| **Net fitness effect** | **Calculation** | **Mean fitness change in fertility given as percentage points** | | |
| --- | --- | --- | --- | --- |
| *Immune costs* | mean of costs #5,#6,#7,#8 | | 4.78 |  |
| A sperm, all | mean of costs #5,#6 | | 5.44 |  |
| B sperm, all | mean of costs #7,#8 | | 4.13 |  |
| Direct (averaged for B sperm, A sperm) | mean of cost #6,#8 | | 1.79 |  |
| Indirect (averaged B sperm, A sperm) | mean of costs #5,#7 | | 7.78 |  |
| Sperm storage benefit (A sperm) | cost #6-#5 | | -2.10 |  |
| Sperm storage benefit (B sperm sperm) | cost #8-#7 | | -9.89 |  |
| Net costs on sperm (averaged for B sperm, A sperm) | mean of costs (#6-#5),(#8-#7) | | -6.00 |  |
| Mean costs on sperm (averaged for B sperm, A sperm) | mean of costs #5,#6,#7,#8 - mean of (#6-#5),(#8-#7) | | 10.78 |  |
| *Microbial costs* | mean of costs #9,#10,#11,#12 | | 17.91 |  |
| A sperm, all | mean of costs #9,#10 | | 4.65 |  |
| B sperm, all | mean of costs #11,#12 | | 31.18 |  |
| Direct (averaged for B sperm, A sperm) | mean of costs #10,#12 | | 25.27 |  |
| Average indirect microbe effects (B sperm, A sperm) | mean of costs #9,#11 | | 10.56 |  |
| Sperm storage cost of co-exposed sperm | cost #10-#9 | | 4.12 |  |
| Sperm storage cost of B sperm | cost #12-#11 | | 25.29 |  |
| Net microbial sperm damage (averaged across B sperm, A sperm) | mean of costs (#10-#9),(#12-#11) | | 14.71 |  |
| Mean microbe costs on sperm | mean of costs #9,#10,#11,#12 - mean of costs (#10-#9),(#12-#11) | | 3.21 |  |
| **Net cost of microbial damage to B sperm (compared to A sperm)** | cost (#12-#11)-(#10-#9) | | **21.17** |  |

**Table S3** Model selection of female fecundity as a function of treatment level. Models are sorted in ascending order by their AICc values. The models with the highest support from the data, as indicated by their AICc weights, were models separating control, mating with foreign male, wounding and female immune system effects from sperm coadaptation level and sperm-microbe contact.

| **Model** | | | **Treatment levels** | **df** | **logLik** | **AICc** | **deltaAICc** | **weight** |
| --- | --- | --- | --- | --- | --- | --- | --- | --- |
| 10 | (C+F+W+ISIN+ISD+ MIA+MDA), (**MIB+MDB)** | | 3 | -1114.793 | 2235.704 | 0.000 | 0.323 |  |
| 7 | (C+F+W+ISIN+ISD+MIA+MDA), **MIB, MDB** | | 4 | -1114.117 | 2236.433 | 0.728 | 0.224 |  |
| 8 | (C+F+W+ISIN+ISD+MIA+MDA+MIB), **MDB** | | 3 | -1115.344 | 2236.805 | 1.101 | 0.186 |  |
| 9 | (C+F+W+ISIN+ISD), **(MIA+MDA),(MIB+MDB)** | | 4 | -1114.793 | 2237.784 | 2.080 | 0.114 |  |
| 6 | (C+F+W+ISIN+ISD+MIAA), **MDA, MIB, MDB** | | 5 | -1114.117 | 2238.533 | 2.829 | 0.078 |  |
| 5 | (C+F+W+ISIN+ISD), **MIA, MDA, MIB, MDB** | | 6 | -1114.117 | 2240.654 | 4.950 | 0.027 |  |
| 11 | (C+F+W+ISIN+ISD), (**MIA+MDA+MIB+MDB**) | | 3 | -1117.489 | 2241.096 | 5.392 | 0.022 |  |
| 4 | (C+F+W+ISIN), **ISD, MIA, MDA, MIB, MDB** | | 7 | -1114.056 | 2242.676 | 6.972 | 0.010 |  |
| 3 | (C+F+W), **ISIN, ISD, MIA, MDA, MIB, MDB** | | 8 | -1113.210 | 2243.148 | 7.444 | 0.008 |  |
| 2 | (C+F), **W, ISIN, ISD, MIA, MDA, MIB, MDB** | | 9 | -1112.392 | 2243.699 | 7.994 | 0.006 |  |
| 1 | **C, F, W, ISIN, ISD, MIA, MDA, MIB, MDB** | | 10 | -1112.392 | 2245.907 | 10.203 | 0.002 |  |

C = Control; F = Mating with foreign male; W = Wounding; ISIN = Indirect effect of female immune system activation; ISD = Direct effect of female immune system activation; MIA = Microbial effects indirect on A sperm; MDA = Microbial effects direct on A sperm; MIB = Microbial effects indirect on B sperm; MDB = Microbial effects direct on B sperm

**Data S1 – semen microbe adaptation.csv (separate file)**

Complete data set for the model selection and survival analyses on the probability of laying unfertilized eggs and sperm function loss in this study.

Readme tab for columns in data file:

female = female ID

treatment.levels = treatments levels used for calculation of relative fitness reduction

sperm = sperm genotype/male population

stab.time.after.mating = time of microbial challenge treatment

microbe.contact = direct vs. indirect sperm-microbe contact

stab.type = type of challenge

model1 to 11 = different treatment combinations for model selection

female.pronotum.size = female pronotum size in mm

male.pronotum.size = male pronotum size in mm

total.fertile.eggs = total number of fertile eggs laid over 10 weeks

total.infertile.eggs = total number of infertile eggs laid over 10 weeks

total.eggs = total number of eggs laid over 10 weeks

survival = survival in days

event = 1 female died / 0 female did not die

time.to.infertile = number of days until second infertile egg was laid

infertile = 1 female laid at least 2 infertile eggs / 0 female did not lay infertile eggs

fertile.eggs.week1 to 10 = fertile eggs laid per week; week 1 to 10

infertile.eggs.week1 to 10 = infertile eggs laid per week; week 1 to 10

total.eggs.week1 to 10 = total number of eggs laid per week; week 1 to 10
